# Network Meta-Analysis with Class Effects: A Practical Guide and Model Selection Algorithm

**DOI:** 10.1177/0272989X251389887

**Published:** 2025-11-08

**Authors:** Samuel J. Perren, Hugo Pedder, Nicky J. Welton, David M. Phillippo

**Affiliations:** School of Mathematics, University of Bristol, Bristol, UK; Bristol Medical School (Population Health Sciences), University of Bristol, UK; Bristol Medical School (Population Health Sciences), University of Bristol, UK; Bristol Medical School (Population Health Sciences), University of Bristol, UK

**Keywords:** Bayesian evidence synthesis, class effects, hierarchical models, model selection strategy, network meta-analysis

## Abstract

Network meta-analysis (NMA) synthesizes data from randomized controlled trials to estimate the relative treatment effects among multiple interventions. When treatments can be grouped into classes, class effect NMA models can be used to inform recommendations at the class level and can also address challenges with sparse data and disconnected networks. Despite the potential of NMA class effects models and numerous applications in various disease areas, the literature lacks a comprehensive guide outlining the range of class effect models, their assumptions, practical considerations for estimation, model selection, checking assumptions, and presentation of results. In addition, there is no implementation available in standard software for NMA. This article aims to provide a modeling framework for class effect NMA models, propose a systematic approach to model selection, and provide practical guidance on implementing class effect NMA models using the multinma R package. We describe hierarchical NMA models that include random and fixed treatment-level effects and exchangeable and common class-level effects. We detail methods for testing assumptions of heterogeneity, consistency, and class effects, alongside assessing model fit to identify the most suitable models. A model selection strategy is proposed to guide users through these processes and assess the assumptions made by the different models. We illustrate the framework and structured approach for model selection using an NMA of 41 interventions from 17 classes for social anxiety.

**Highlights:**

- Provides a practical guide and modelling framework for network meta-analysis (NMA) with class effects.
- Proposes a model selection strategy to guide researchers in choosing appropriate class effect models.
- Illustrates the strategy using a large case study of 41 interventions for social anxiety.

Network meta-analysis (NMA) is an evidence synthesis method that combines the summary treatment effects published from randomized controlled trials (RCTs) to derive pooled estimates of relative treatment effects between multiple interventions.^1,2^ This approach is particularly valuable in health care decision making, in which reliable estimates of the effectiveness and cost-effectiveness of treatments are crucial.^3^ NMA coherently combines the relevant data on each comparison of interest, which includes direct evidence from head-to-head trials and indirect evidence via connected paths of study comparisons.^4,5^ NMA respects randomization within studies, thus maintaining the validity of within-study comparisons.^6,7^

In some evidence networks, there may be many distinct treatments, and these may be categorized into treatment classes. In this situation, those interpreting the results from the NMA may want to do so either for specific treatments or for treatment classes. Another challenge when there are many distinct treatments is that each treatment comparison may be informed by relatively few trials. This scenario often leads to data sparsity, which poses a risk of substantial parameter uncertainty in the analysis,^8,9^ or even a disconnected network, in which case comparisons cannot be made at all between disconnected treatments. To mitigate these issues, the NMA approach can be extended to include class effects with hierarchical models that enable the “borrowing of strength” between treatments belonging to the same class.^1,10,11^ In addition, class models facilitate the ability to make recommendations at the class level, which can be particularly useful in clinical guideline development, where it may be desirable to recommend an entire class of interventions.

Hierarchical models are routinely used in pairwise meta-analysis and NMA to allow for between-study heterogeneity in treatment effects. When treatments can be grouped into classes, an additional hierarchical level can be introduced into the model, where treatment effects within a class come from a common distribution of effects with a class mean effect.^9,12^ For example, a class might consist of drugs that share the same mechanism of action, such as beta-blockers used in the treatment of hypertension, which, despite having different individual characteristics, are expected to exert their effects in a similar manner and so can reasonably be expected to have similar relative effects around a class mean. Incorporating classes into the NMA model allows information sharing within intervention classes, which may improve the precision of treatment effect estimates where there are sparse data. Class effects models can also allow for the estimation of treatment and class effects in networks that are disconnected at the treatment level but connected at the class level. Moreover, class effects models can support health care decision making by providing an understanding of treatment efficacy within and across different classes.

Despite the potential of NMA class effects models and numerous applications in a wide range of disease areas,^13–15^ the literature lacks a comprehensive guide setting out the range of possible class effect models, their assumptions and how these are assessed, practical considerations for model estimation, and presentation of results. Fitting class effects models has also required analysts to rely on bespoke modeling code, as these models have not yet been implemented in a user-friendly software package. Furthermore, there is a real need for a systematic approach to selecting an appropriate class effect model for a specific dataset, helping analysts to navigate and assess the inherent assumptions associated with these models.

In this article, we provide a detailed practical guide on the use of class effect models and propose an approach for model selection to assess their assumptions and identify an appropriate class model. We describe how these models and processes are implemented in the multinma R package,^16^ which we have updated with new functionality for class effects NMA models. However, the guidance and model selection process may be followed by analysts fitting these models in other statistical software.

We begin by describing a motivating example of treatments for social anxiety disorder. We then outline the various modeling choices available for incorporating class effects into NMA and the underlying assumptions that they make and present methods to assess these assumptions. We then propose a model selection strategy and apply this to the social anxiety example. We show how treatment effect estimates are altered when incorporating class effects with forest plots. Finally, we discuss the implications of incorporating class effects in NMA and the implementation of class effects within the multinma R package.

## Example: Social Anxiety

**Figure 1:**
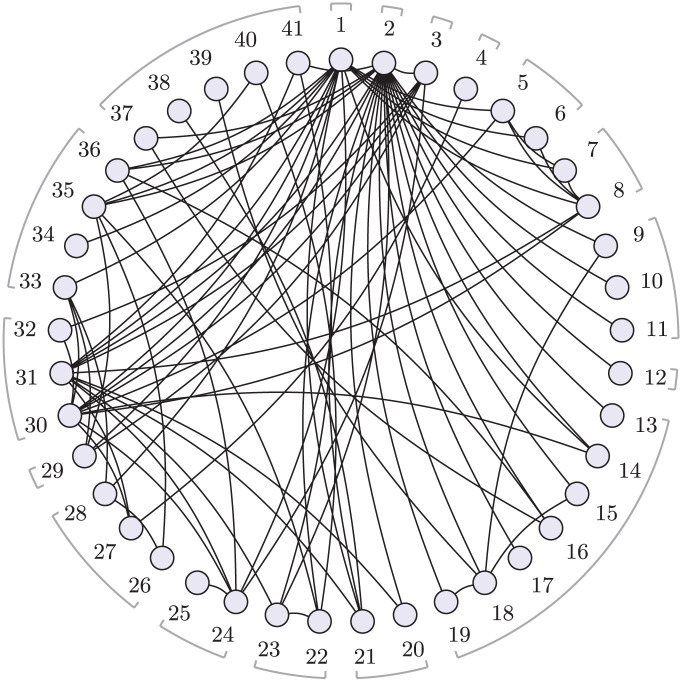
Social anxiety treatment network. Circles (nodes) represent treatments, and connecting lines show study comparisons. Numbers around the edge are the treatment codes. Treatment classes are indicated by brackets; some classes contain a single treatment. See Supplementary Table S4 for treatment codes. Reproduced from Phillippo et al.^18^ licensed under CC BY 4.0.

**Figure 2:**
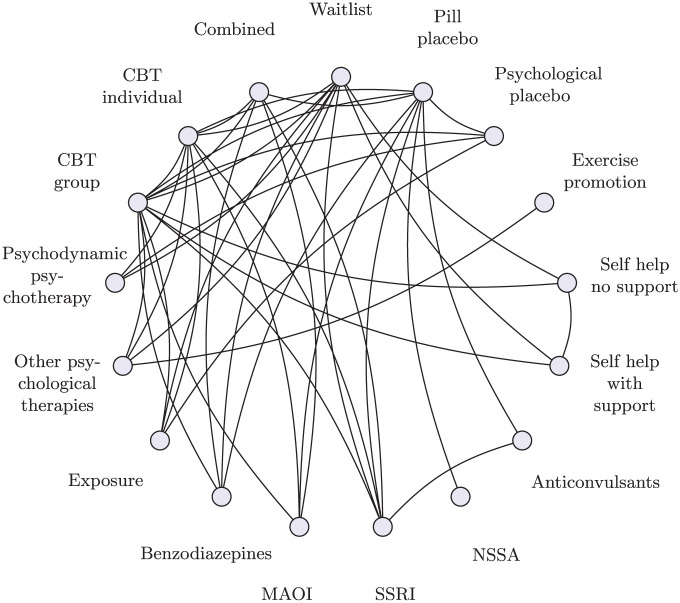
Social anxiety class network. Circles (nodes) represent classes, and connecting lines show study comparisons.

To illustrate the methods, we use an NMA of first-line treatments for social anxiety disorder in adults.^17^ A systematic review identified 101 clinical trials with a total of 13,164 participants, comparing 41 different treatments, which are further categorized into 17 distinct classes. Direct comparisons are visualized in Figure 1 at the treatment level and Figure 2 at the class level. The interventions of interest include oral drugs, psychological or behavioral therapies, and combinations of pharmacologic and psychological therapies. We follow Mayo-Wilson et al.^17^ by analyzing results as standardized mean differences (SMDs), allowing comparison despite the diversity of outcome scales across studies.

## Class Effects Framework for NMA

We now set out the mathematical framework for NMA, before extending this to incorporate a range of class effects models.

### NMA Model

Given summary outcomes $y_{\mathrm{jk}}$ on treatment k in study j , the standard NMA model can be written as

(1) $$y_{\mathrm{jk}}\sim \pi \left(\theta_{\mathrm{jk}}\right)$$

(2) $$\begin{array}{c} g\left(\theta_{\mathrm{jk}}\right)=\mu_{j}+\delta_{\mathrm{jk}} \end{array}$$

where π(·) is a suitable likelihood. $\theta_{\mathrm{jk}}$ is the expected summary outcome on treatment k in study j , which is transformed onto the linear predictor scale with a suitable link function g(·) . $\mu_{j}$ are study-specific intercepts, and $\delta_{\mathrm{jk}}$ is the study-specific relative effect of treatment k versus the network reference treatment 1. For example, with a continuous outcome reported in each arm, it is common to use a normal likelihood and the identity link function, so

(3) $$\begin{array}{c} y_{\mathrm{jk}}\sim N\left(\theta_{\mathrm{jk}},s_{\mathrm{jk}}^{2}\right) \end{array}$$

(4) $$\theta_{\mathrm{jk}}=\mu_{j}+\delta_{\mathrm{jk}}$$

where $s_{\mathrm{jk}}$ is the standard deviation of the summary outcome on treatment k in study j .

In a random effects (RE) NMA, there is a hierarchical model in which study-specific relative effects $\delta_{\mathrm{jk}}$ follow a common distribution:

(5) $$\begin{array}{cc} \delta_{\mathrm{jk}}\sim N\left(d_{k},\tau^{2}\right) & \; \end{array}$$

where $\tau^{2}$ is the between-study variance, assumed to be common across comparisons, and $d_{1k}$ is the pooled mean treatment effect for treatment k relative to treatment 1, which we denote $d_{k}$ for ease of notation. We set $\delta_{j1}=d_{1}=0$ for the reference treatment 1. Relative effects $d_{\mathrm{ab}}$ between any 2 treatments a,b∈{2,…,K} satisfy the consistency equations $d_{\mathrm{ab}}=d_{1b}-d_{1a}=d_{b}-d_{a}$ , allowing 2 treatments to be compared even if not included in the same head-to-head trial.

In this parameterization, in which treatment 1 serves as the reference for the entire network, every nontreatment 1 arm is given a random effect $\delta_{\mathrm{jk}}$ . Studies with more than 1 nontreatment arm therefore have multiple RE, which are correlated and modeled using a multivariate normal distribution. The assumption that the heterogeneity variance $\tau^{2}$ is common across all pairwise comparisons leads to correlation $\mathrm{corr}\left(\delta_{\mathrm{ja}},\delta_{\mathrm{jb}}\right)=0.5$ between the RE in each study j for any treatment a,b>1 .^2^

In a fixed effect (FE) NMA, the study-specific relative effects $\delta_{\mathrm{jk}}$ are set equal to $d_{k}$ , which is equivalent to assigning a value of zero to $\tau^{2}$ in equation 5.

Within the Bayesian framework, we assign prior distributions to the parameters being estimated. For these standard NMA models, we require prior distributions for $d_{k}$ and (for an RE model) τ .

### Class Effects Models

The NMA model can be extended to incorporate class effects in several ways, each making different assumptions about the treatment effects within classes. We will refer to the “standard NMA model” as the “no class NMA model” from this point forward.

#### Exchangeable class effects

Although treatments within the same class may share similarities, factors such as bioavailability and tolerability can lead to variability in their relative effectiveness. In such cases, or when there is clinical uncertainty regarding the degree of similarity between treatments, an exchangeable class effects model may be suitable.

In an exchangeable class effects model, the treatment effect $d_{k}$ for a specific treatment k follows a normal distribution, with parameters defined by the class $c_{k}$ to which the treatment belongs:

(6) $$\begin{array}{cc} d_{k}\sim N\left(m_{c_{k}},\sigma_{c_{k}}^{2}\right) & \; \end{array}$$

where $m_{c_{k}}$ represents the pooled mean effect of all treatments within class $c_{k}$ , drawing the individual treatment effects toward this pooled class effect. The standard deviation $\sigma_{c_{k}}$ quantifies the variation of the treatment effects within the class.

In the Bayesian framework, we require prior distributions for the class mean effects $m_{c}$ and the class-specific standard deviations $\sigma_{c}$ .

The class effects distribution (equation 6) allows for the sharing of information on relative effects across treatments in the same class. The resulting treatment effect $d_{k}$ is a shrunken estimate in the sense that it will be drawn toward the class mean $m_{c}$ . The amount of shrinkage depends on both the precision of $d_{k}$ and the between-treatment variance $\sigma_{c}^{2}$ . In a class with low between-treatment variance ( $\sigma_{c}^{2}$ ), imprecise treatment effect estimates that differ substantially from the class mean $m_{c}$ are pulled strongly toward it, whereas shrinkage will be reduced for treatment effects that are estimated more precisely. The exchangeable class structure can either increase or decrease the precision of $d_{k}$ . Precision can decrease when the class-level parameters are themselves uncertain, for example, if $\sigma_{c}^{2}$ is large, because the additional uncertainty from estimating $m_{c}$ and $\sigma_{c}^{2}$ can outweigh the gains from pooling.

In some cases, class effect models can also facilitate the estimation of relative effects in evidence networks that are disconnected at the treatment level, as long as they are connected at the class level.

Note that class effects assumptions apply to the relative effects of treatments; therefore, in the exchangeable class effects model, the reference treatment is always a stand-alone treatment and not modeled as part of a class. The classes are defined for all the nonreference treatments. This approach ensures the reference treatment serves as a clear baseline, allowing all other treatment and class effects to have a straightforward interpretation relative to this baseline.

#### Common class effects

For treatments within a class that are expected to have highly similar effects, a common class effect model may be appropriate.

The common class effects model assumes that all treatments within the class have identical treatment effects on the outcome of interest. Mathematically, this is represented by setting $d_{k}=m_{c_{k}}$ or equivalently by setting the class variance parameters $\sigma_{c}^{2}$ to zero. When conducting an NMA with common class effects, individual treatment effects are not distinguished unless the treatment is the only treatment within the class, in which case the class effect is equal to the effect of that single treatment.

When we have random treatment effects within a common class model, the study-specific relative effects (equation 5) are written as

(7) $$\begin{array}{cc} \delta_{\mathrm{jk}}\sim N\left(m_{c_{k}},\tau^{2}\right), & \; \end{array}$$

and when we have fixed treatment effects within a common class model, the linear predictor (equation 2) is replaced with

(8) $$\begin{array}{cc} g\left(\theta_{\mathrm{jk}}\right)=\mu_{j}+m_{c_{k}}. & \; \end{array}$$

In contrast, within common class effect models, the reference treatment is included as part of its respective class because the model assumes that the relative effects of treatments in the same class are identical. The reference treatment may be a single treatment or included as part of a class. In the latter case, comparisons are made relative to the entire reference class rather than an individual reference treatment.

#### Further variations

In equation 6, we allow a different class standard deviation $\sigma_{c}$ for each class. However, there may be insufficient information to estimate variance parameters for classes with only a small number of treatments, and for classes with only 1 treatment, these cannot be estimated at all. To address this, class standard deviations can be shared within a group of 2 or more classes, so that $\sigma_{c_{k}}$ in equation 6 becomes $\sigma_{Q_{c_{k}}}$ where *Q* is the group of classes. By grouping class standard deviations, we are able to produce reasonable variance estimates for classes with few treatments and produce estimates for classes with only 1 treatment. Before combining class standard deviations, one should consult a clinical expert to verify that the classes to be combined are expected to have similar standard deviations.

In practice, it may be necessary or desirable to combine different types of class assumptions for different classes within a single model, perhaps for pragmatic reasons due to the data available, or with a specific clinical rationale or decision question in mind. For example, it may be that there is very little variation in effects within pharmacologic classes, and a common class model is appropriate; equations 7 or 8 are used for those treatments/classes, but for psychological classes, an exchangeable class model is required, and equation 6 is used for those treatments/classes. Such a setup, in which some classes are modeled under 1 assumption (e.g., common class) while others are modeled under a different assumption (e.g., exchangeable class), is often referred to as a mixed-class model. Mathematically, a mixed-class model is designed to allow different assumptions to be made about different classes in the network, including the assumption of no class effects (i.e., independent treatment effects) for given treatments. This flexibility enables a model that can be adapted to the specific needs of each class, which may vary depending on statistical considerations, clinical relevance, or pragmatic constraints due to available data.

### Model Comparison and Assessing Assumptions

It is important to assess the validity of the assumptions made by NMA and the different class effects models. We achieve this by comparing the fit of models making different assumptions, using the posterior mean residual deviance, the deviance information criterion (DIC), and variance parameters in the hierarchical models. The residual deviance evaluates the fit of the model by measuring the discrepancies between the observed and predicted values, with lower values of residual deviance indicating a better fit of the model.^1^ The DIC penalizes the residual deviance by the effective number of parameters ( $p_{D}$ ), which quantifies the complexity of the model by considering the influence of the parameters on the fit. We prefer models with lower posterior mean residual deviance and DIC, in which differences of more than 3 to 5 points in the DIC value are considered statistically meaningful.^19^

#### Assessing heterogeneity

To assess heterogeneity, we compare the fit of models with fixed and random treatment effects. In a RE model, the between-study standard deviation τ quantifies heterogeneity, where lower values of τ indicate less heterogeneity. The between-study standard deviation is evaluated on the same scale as the relative treatment effects. Typically, an RE model will give a better fit based on residual deviance as compared with an FE model because it can account for heterogeneity between the results of different studies by increasing τ .^1^ In model selection, it is essential to check the estimated τ as well as posterior mean residual deviance and DIC statistics. If the DIC of the FE and RE models is similar, the FE model may be favored due to its simpler interpretation.

#### Assessing inconsistency

Inconsistency in NMA occurs when the direct evidence and indirect evidence are not in agreement with each other. We assess this by comparing the unrelated mean effects (UME) inconsistency model with the standard no-class NMA (consistency) model.

The UME model relaxes the assumption of consistency by estimating a separate treatment effect for each pair of treatments compared in the network exclusively using direct evidence.^20^ The UME model is analogous to performing individual pairwise meta-analyses for each treatment comparison, with the exception that for an RE model, the between-study variance is jointly estimated across all comparisons.

For the UME model, the linear predictor equation 2 is replaced by

(9) $$\begin{array}{c} g\left(\theta_{\mathrm{jk}}\right)=\mu_{j}^{\left(t_{j1}\right)}+\delta_{jt_{j1}k} \end{array}$$

where $t_{j1}$ is the treatment in arm 1 of study j . Under the UME model, the study baseline $\mu_{j}^{\left(t_{j1}\right)}$ is now with respect to the treatment $t_{j1}$ in arm 1 of each study, rather than the network reference treatment 1. Model 9 represents a UME RE model; however a UME FE model can be fitted in the same way as the no-class NMA model, by replacing $\delta_{jt_{j1}k}$ with $d_{t_{j1}k}$ . For both the FE and RE UME models, each of the relative effects $d_{\mathrm{ab}}$ for all a,b∈{1,…,K;b>a} are given independent prior distributions, such that no consistency relationship is imposed.

The no-class NMA and UME models can be compared using the posterior mean residual deviance and DIC, with substantially better fit for the UME model suggesting that globally direct evidence and indirect evidence are not in agreement in the network and there is evidence of inconsistency. In an RE model, we should also compare estimates of between-study heterogeneity: a reduction in the between-study standard deviation in the UME model may indicate the presence of inconsistency.^1^ It is important to recognize that even if the total residual deviance or DIC values are similar between the no-class NMA and UME models, a reduction in between-study standard deviation can still signify inconsistency.^21^

Global tests can, however, sometimes fail to detect important local inconsistencies that can balance each other out when assessed globally. Local inconsistency can be explored by comparing the contribution of each data point to the total posterior mean residual deviance between models, using a deviance contribution plot (dev-dev plot),^1^ as this can help identify individual data points that contribute to inconsistency. With residual deviances from the no-class NMA model plotted on the x -axis and residual deviances from the UME model on the y -axis, data points that fall below the line of equality show that the UME gives a better fit than the no-class NMA model for that data point, suggesting it may be inconsistent with other data points in the network. Any data points that are notably below the line of equality should be investigated further by node splitting, which can be used to obtain direct and indirect estimates and perform a local test for inconsistency.^1,21^ The data should also be checked for potential data extraction errors and study-specific features (e.g., differences in population, intervention, or outcome definition or risk-of-bias assessments) that may be contributing to the inconsistency.

#### Assessing class model assumptions

To assess the statistical validity of different class assumptions, we can compare model fit statistics and between-study and between-treatment standard deviation parameters for the different models. We can produce dev-dev plots to help identify data points that may not align with the global class assumption. When comparing models, we place the model with the stronger assumption on the x -axis. For example, in a comparison between the no-class and exchangeable class models, the exchangeable model is on the x -axis and the no-class model on the y -axis. Data points that fall below the equality line suggest a better fit for the no-class model, while those above favor the exchangeable model. Similarly, when comparing the common and exchangeable models, residuals from the common model are plotted on the x -axis and those from the exchangeable model on the y -axis. Points below the line indicate a better fit to the exchangeable model and points above to the common model. Substantial deviations from the line of equality may warrant further investigation.

If exchangeable class models are being evaluated, we can inspect the class standard deviation parameters ( $\sigma_{c}$ ) to assess the homogeneity of treatment effects within each class. We can use this along with the dev-dev plot to identify any classes that may not align well with the global class assumption. If the assumptions underlying the class effect are not valid, modeling class effects may introduce additional heterogeneity or inconsistency when compared with the standard no-class model. However, the range of different modeling options, as summarized in the “Class Effects Models” section, means that model selection is not straightforward. In the next section, we set out a structured model selection strategy to guide analysts in choosing between the different models.

#### Assessing model fit

Leverage plots^19^ provide a visual approach to identify influential and/or poorly fitting observations. Leverage quantifies the degree to which each data point influences the estimated model parameters. The sum of individual leverages equals the effective number of parameters ( $p_{D}$ ).^1^ The vertical axis of the plot represents leverage, while the horizontal axis corresponds to the square root of the deviance. Overlaid are contours of the form $x^{2}+y=c$ for c=1,2,3 which indicate curves of constant contribution to the DIC.^19^ Identifying points with high leverage is important because they can have a considerable effect on model complexity and fit. For multiarm studies with contrast data, standardized residual deviances and leverages are plotted by dividing through by the degrees of freedom (number of arms minus one). This adjustment ensures consistent interpretation across studies and data types. The leverage for each data point is calculated as the posterior mean of the residual deviance minus the deviance at the posterior mean of the parameters.^1^ A high leverage means that removing or altering this data point would have a notable effect on the parameter estimates. Points beyond the c=3 curve typically indicate poor model fit and highlight areas where the model struggles to represent the data accurately. These points should be investigated to understand their impact and validity. To address data points contributing to poor model fit, the accuracy of the data should be verified and the study characteristics assessed to determine if they differ substantially from others. It is crucial to understand why they contribute to poor model fit, ensuring clear justification for their inclusion and evaluating their effect on the results.

## Model Selection Process

**Figure 3:**
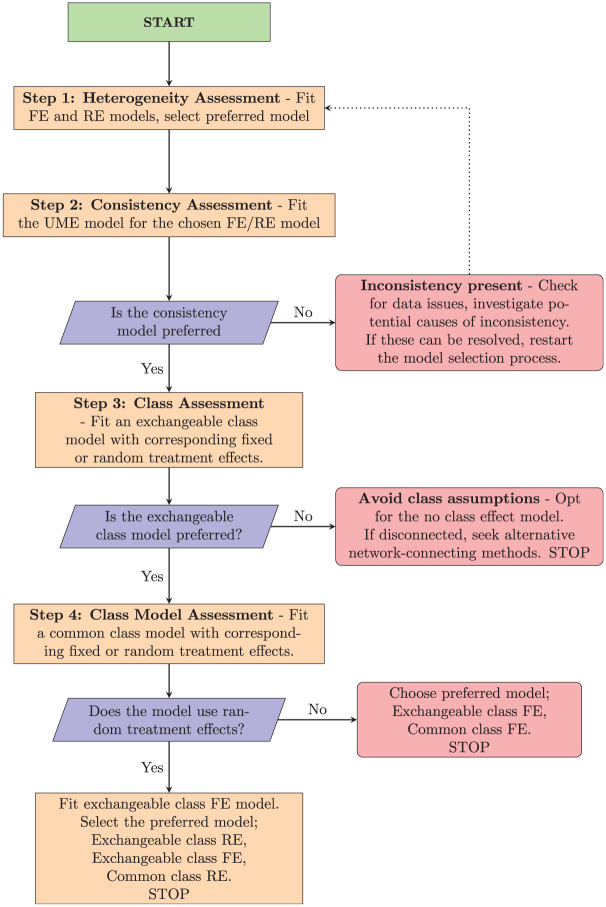
Flowchart for proposed model selection process.

The class effects NMA framework encompasses a range of potential models and assumptions produced by different combinations of modeling choices for consistency, heterogeneity, and class effects (Table 1). To help analysts choose between candidate models and assess assumptions in a principled and structured manner, we propose a model selection process given by the flow chart in Figure 3, described and discussed below. Prior to commencing this process, it should be ensured that treatments have been assigned classes and considered whether any classes will share class standard deviations for clinical reasons and/or due to containing only a small number of treatments. The process begins with assessing heterogeneity and inconsistency for the standard no-class model. If there is no evidence of inconsistency, then we proceed to explore the validity of the different class model assumptions. Throughout this process, we retain the full dataset in every model. Arms or studies are never combined or dropped (even when comparisons occur only within a class), so residual deviance, *p_D_*, and DIC are directly comparable across all models. Note that if the evidence network is disconnected at the treatment level (but connected at the class level), then the standard no-class model cannot reliably estimate the relative effects between all treatments, making it unsuitable as a final model for decision making.

**Table 1:** Eight Models with Corresponding Assumptions and Algebraic Formulations for Use in Our Proposed Model Selection Strategy

Model Name	Class Effect	Heterogeneity	Consistency	Algebra
UME RE	No class (independent)	Random	Inconsistent	$g\left(\theta_{\mathrm{jk}}\right)=\mu_{j}^{\left(t_{j1}\right)}+\delta_{jt_{j1}k}$ $\delta_{jt_{j1}k}\sim N\left(d_{t_{j1}k},\tau^{2}\right)$
UME FE	No class (independent)	Fixed	Inconsistent	$g\left(\theta_{\mathrm{jk}}\right)=\mu_{j}^{\left(t_{j1}\right)}+d_{t_{j1}k}$
No class RE	No class (independent)	Random	Consistent	$g\left(\theta_{\mathrm{jk}}\right)=\mu_{j}+\delta_{\mathrm{jk}}$ $\delta_{\mathrm{jk}}\sim N\left(d_{k},\tau^{2}\right)$
No class FE	No class (independent)	Fixed	Consistent	$g\left(\theta_{\mathrm{jk}}\right)=\mu_{j}+d_{k}$
Exchangeable class RE	Exchangeable	Random	Consistent	$g\left(\theta_{\mathrm{jk}}\right)=\mu_{j}+\delta_{\mathrm{jk}}$ $\delta_{\mathrm{jk}}\sim N\left(d_{k},\tau^{2}\right)$ $d_{k}\sim N\left(m_{c_{k}},\sigma_{c_{k}}^{2}\right)$
Exchangeable class FE	Exchangeable	Fixed	Consistent	$g\left(\theta_{\mathrm{jk}}\right)=\mu_{j}+d_{k}$ $d_{k}\sim N\left(m_{c_{k}},\sigma_{c_{k}}^{2}\right)$
Common class RE	Common	Random	Consistent	$g\left(\theta_{\mathrm{jk}}\right)=\mu_{j}+\delta_{\mathrm{jk}}$ $\delta_{\mathrm{jk}}\sim N\left(m_{c_{k}},\tau^{2}\right)$
Common class FE	Common	Fixed	Consistent	$g\left(\theta_{\mathrm{jk}}\right)=\mu_{j}+m_{c_{k}}$

FE, fixed effects; RE, random effects; UME, unrelated mean effects.

### Step 1: Heterogeneity Assessment

The first step of our proposed model selection process is to assess heterogeneity in the no-class model. We do this by comparing model fit metrics for the FE no-class model with the RE no-class model. This evaluation helps determine whether a fixed or RE model is more appropriate at the treatment level. If both models produce similar model fit, we might prefer the FE model, as it is the simpler of the 2 models, making the results easier to interpret.^1^ If the network is disconnected at the treatment level, we still conduct this heterogeneity assessment; the comparisons of model fit are still valid, even though neither model can estimate a full set of relative effects for decision making.

### Step 2: Consistency Assessment

Once the initial heterogeneity model is selected, we proceed to assess treatment-level inconsistency by fitting a UME model with the same fixed or RE as the preferred no-class model from step 1. This step follows the inconsistency-checking process outlined in the “Assessing Inconsistency” section covering treatment-level consistency checking. We compare residual deviance, DIC, and τ between the no-class and UME models to assess global inconsistency and examine a dev-dev plot to identify any specific data points that are suggestive of inconsistency. Node splitting may be conducted for any treatment comparisons identified from the dev-dev plots. If evidence of inconsistency is identified, the data should first be checked for potential data extraction errors and the studies then examined for factors that may explain the inconsistency such as population characteristics, intervention definitions, outcome definitions, or study design. It may be necessary to reassess the research objectives and revise the inclusion and exclusion criteria for the studies in the network with respect to any such identified factors to resolve inconsistency; additional strategies could include analyzing subgroups or applying meta-regression techniques.^20^

If inconsistency cannot be resolved, it might indicate that a complete analysis using the current data is unfeasible.

### Step 3: Class Assessment

If there is no evidence of inconsistency, the next step is to identify whether a class model is suitable for the data. Here, we compare only the exchangeable class model with corresponding fixed or RE against the standard no-class model selected at step 1. This is because if the exchangeable class model indicates lack of fit, then the common class model that makes stronger assumptions would exhibit similar or more severe issues. Again, we can use the residual deviance, DIC, and between-study standard deviation τ as well as the dev-dev plot to assess if including class effects in the model is reasonable. If we find that the no-class model is preferred over the exchangeable class effect model, then it may not be appropriate to make any class effects assumption in the data. If the network is disconnected at the treatment level but a class model assumption is not supported, we may need to look for alternative methods to achieve a connected network (for example, see Stevens et al.^22^). If the exchangeable class model is preferred over the no-class model, the next step is to proceed to the class model assessment (step 4).

### Step 4: Class Model Assessment

In this step, we assess which form of class effects model is most appropriate for the data. In this final step, we have 2 possible sets of models to compare against each other in order to select our final preferred model, which depend on whether FE or RE were selected in step 1. If we selected an RE model in step 1, then it is possible that the between-study heterogeneity could be explained by variation between treatments within classes. Therefore, we should compare the exchangeable class RE model, the common class RE model, and the exchangeable class FE model. Based on model fit metrics (residual deviance, DIC, τ , dev-dev plots, and class standard deviation), the preferred model is our final selected model. If we selected a FE model at step 1, then we need to compare only the exchangeable class FE model and the common class FE model. Based on model fit metrics (residual deviance, DIC, τ , dev-dev plots, and class standard deviation), the preferred model is our final selected model. If the model fit metrics are indifferent between models, we would favor the model that is simplest and most interpretable for the decision maker (typically the model with the fewer parameters). Analysts should justify their final choice explicitly.

## Class Models in multinma

We updated the multinma R package in version 0.8.0 to support fitting the class effect models outlined in Table 1. In particular, we introduced new arguments to the nma() function, namely, class_effects, class_sd, prior_class_mean, and prior_class_sd. A comprehensive vignette (https://dmphillippo.github.io/multinma/articles/example_social_anxiety.html) illustrates the new class effect functionality with sample code and a recommended workflow. The exact code used in this article is available at https://github.com/sjperren/NMA_with_class_effects.

## Application to the Social Anxiety Example

All analyses were performed using R version 4.2.2^23^ and the multinma package,^16^ which estimates models using Markov chain Monte Carlo simulations in Stan.^24^ We illustrate the different class models by fitting the no class effect, exchangeable class, and common class models, all with RE, to the social anxiety data. We report treatment effect estimates visualized through forest plots, and rank probability plots for treatments and classes, to illustrate how different class assumptions affect treatment effect estimates and rankings. We then demonstrate our model selection strategy applied to the social anxiety data, describing the strategy and decision-making process at each stage.

### Priors and Class SD Sharing

In the standard no-class model, we assign vague independent normal prior distributions with a mean of 0 and a standard deviation of 100 to each treatment effect $d_{k}$ . For all class models, the class effects means $m_{c}$ are given a vague normal prior distribution with a mean of 0 and a standard deviation of 10.

The between heterogeneity standard deviation is given a weakly informative half-normal prior distribution with a standard deviation of 5. We opt for an informative prior for the class-specific standard deviations ( $\sigma_{c}$ ), setting it as a truncated-normal distribution with mean 0.33 and standard deviation 0.1. This restriction limits variability to a clinically plausible range and facilitates the estimation of standard deviations for classes with a small number of treatments. This follows the approach used by the authors of the social anxiety guideline,^13^ except that we replace their inverse-Gamma prior with an equivalent truncated-normal prior for compatibility with the multinma package. Treatment classes are listed in Supplementary Table S4. We grouped the following classes to share class standard deviations; exercise promotion with self-help no support, SSRI/SNRI with NSSA, and psychodynamic psychotherapy with other psychological therapies.

### Comparison of Different Class Models for Social Anxiety Data

In this section, we illustrate how different class effect assumptions affect treatment and class estimates (and rankings). The summary results comparing relative treatment and class effects across models are provided in Supplementary Table S1. The no-class model estimates a total of 40 treatment effects, whereas the common class model estimates 16 class effects because each treatment effect is assumed identical to the class effect. The exchangeable class model provides estimates of both treatment and class effects, where treatment effects are shrunk toward the class mean effect.

**Figure 4:**
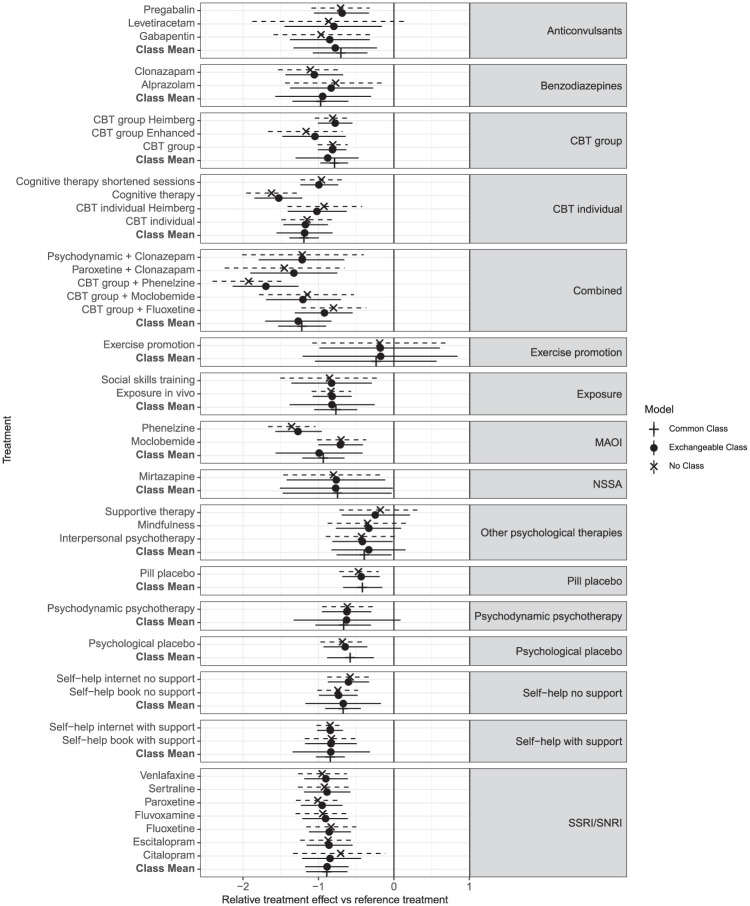
Forest plot showing the relative treatment and class effects against the reference (waitlist) as posterior means and 95% credible intervals, comparing the no-class model to the exchangeable class and common class model, all with random treatment effects. Results are grouped by class.

The forest plot comparing the no-class effect model with both the common and exchangeable class model (Figure 4) shows that the exchangeable class model consistently yields narrower credible intervals (CrIs) than the no-class model does, indicating more precise treatment effect estimates. Also, the treatment effects in the exchangeable class model are shrunk toward their class means. For example, the treatment effect for citalopram in the no-class model is estimated at −0.70 (95% Crl: −1.30 to −0.10), but this is shrunk in the exchangeable class model to −0.84 (95% Crl: −1.22 to −0.44). Similarly, the effect of enhanced CBT changes from −1.17 (95% Crl: −1.65 to −0.66) in the no-class model to −1.05 (95% Crl: −1.47 to −0.64) in the exchangeable class model. The effect of levetiracetam changes from −0.86 (95% Crl: −1.82 to 0.11) in the no-class to a more precise −0.79 (95% Crl: −1.46 to −0.13) in the exchangeable class model, with CrIs that no longer include zero. In this case, the shrinkage in the exchangeable class model leads to more precise estimates of effectiveness compared with the reference treatment. Notice that shrinkage is more pronounced in classes in which there is high between-treatment variability (combined and CBT individual), whereas classes with relatively homogeneous treatment effects to the class mean show minimal movement (SSRI/SNRI).

In classes such as “combined” and “MAOI,” the common class model yields a relatively precise estimate of the class mean, although this may not fully account for variability among individual treatment effects within the class. This limitation becomes evident in the exchangeable model, which produces wider CrIs around the class mean. This increase in interval width stems not only from within-class variation but also from the statistical uncertainty in estimating class standard deviations, especially in classes with limited data. Thus, the common class assumption may not be appropriate for all classes.

Supplementary Figure S1 demonstrates a consistent pattern in treatment rankings in the exchangeable class and no-class models, indicating that rankings at the treatment level are unchanged between these 2 models in this instance.

**Figure 5:**
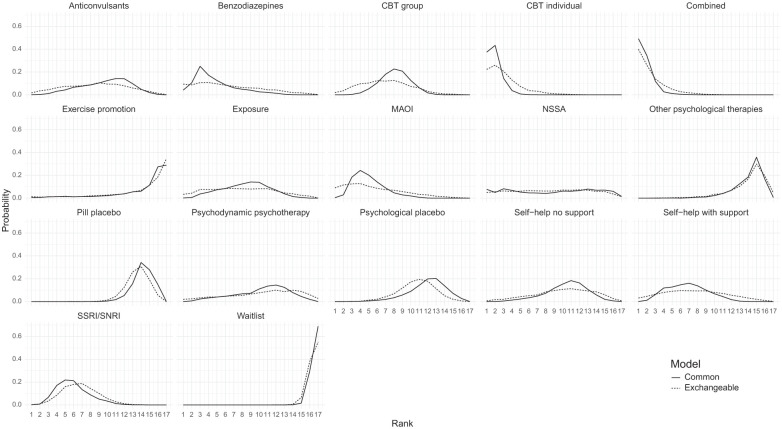
Comparison of rank probabilities of classes between the common class random effects (RE) and exchangeable class RE models.

In Figure 5, a subtle distinction emerges between rankings at the class level for the class effect models. Specifically, the rank distribution plots generated using the common class model exhibit notably higher peaks for certain classes, suggesting a higher level of confidence in the rankings. In contrast, the rank distributions obtained from the exchangeable class model are flatter, signifying a greater degree of uncertainty, shown in Supplementary Figure S3 in the narrower Crls associated with the common class model compared with those of the exchangeable class model.

### Model Selection Using Proposed Strategy

We now apply our proposed model selection strategy to the social anxiety example.

#### Step 1: Heterogeneity assessment

First, we assess heterogeneity by comparing FE and RE of the no-class model using residual deviance, $p_{D}$ and DIC.

Table 2 shows that the DIC for the RE model (257.4) is much lower than that of the FEs model (328.4) due to the large decrease in residual deviance showing a much better fit to the data. Therefore, our preferred no-class model is the RE model, which we use in subsequent steps.

**Table 2:** Residual Deviance, $p_{D}$ and DIC, Deviance Information Criterion Comparison between Fixed Effects and Random Effects Models, with No Class Effects.

Model	Treatment Effects	Residual Deviance	$p_{D}$	DIC
No class effect	Fixed	288.3	40.1	328.4
No class effect	Random	162.5	94.9	257.4

DIC, deviance information criterion.

#### Step 2: Consistency assessment

Next, we assess consistency, comparing model fit statistics for the UME model and the no-class (consistency) model, both with random treatment effects. Table 3 shows the residual deviance is not meaningfully different between the 2 models, suggesting a similar level of model fit between the two. However, the DIC is lower in the no-class model (257.4) compared with the UME model (270.4), because the no-class model is more parsimonious (smaller $p_{D}$ ). Moreover, the between-study standard deviation τ is essentially unchanged between the consistency model (0.21, 95% Crl: 0.15–0.27) and the UME model (0.22, 95% Crl: 0.16–0.29). Altogether, there is no evidence of inconsistency at the global level.

**Table 3:** Residual Deviance, $p_{D}$ , DIC, and Posterior Mean and 95% Credible Interval for τ , for the No-Class and UME Models, Both with Random Effects.

Model	Treatment Effect	Residual Deviance	$p_{D}$	DIC	τ
No class effect	Random	162.5	94.9	257.4	0.21 (0.15–0.27)
UME	Random	161.4	109.1	270.4	0.22 (0.16–0.29)

DIC, deviance information criterion; UME, unrelated mean effects.

**Figure 6:**
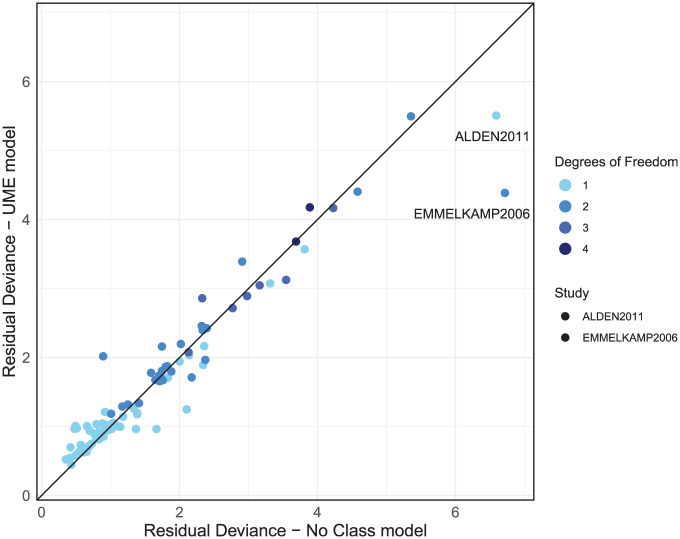
Residual deviance comparison between the unrelated mean effects (UME) and no-class models, both with random effects.

The dev-dev plot (Figure 6) comparing residual deviance contributions for the UME model and no-class model shows that most data points lie close to the line of equality. This suggests a comparable model fit for most data points. We have color-coded the points based on their degrees of freedom, calculated as the number of arms in a study minus one. This highlights that higher residual deviance may be associated with studies containing more arms. However, 2 studies—ALDEN2011 and EMMELKAMP2006—stand out, each with a residual deviance that is smaller in the UME model by a difference of 1 or more compared with the no-class model, as indicated in Figure 6.

We then use node-splitting to investigate direct and indirect treatment effect estimates for the treatments that are within these 2 outlier studies. The ALDEN2011 study reported an SMD of −1.88, with a standard error 0.28 for the “CBT group” against “waitlist.” This effect is substantially larger than that observed in other CBT group trials and deviates from the direct and indirect estimates of −0.89 (CrI −1.16 to −0.63) and −0.73 (CrI −1.01 to −0.44), respectively; however, this is more suggestive of heterogeneity than inconsistency. A review of study characteristics should therefore be completed for ALDEN2011. EMMELKAMP2006 reported an SMD of 0.159 for psychodynamic psychotherapy and −0.06 for CBT individual, which contrasts with both the direct and indirect estimates in this evidence loop: psychodynamic versus waitlist direct and indirect evidence reports SMDs of −0.60 (CrI −0.96 to −0.23) and −0.68 (CrI −1.51 to 0.13), respectively. CBT individual versus waitlist direct and indirect evidence reports SMDs of −0.84 (CrI −1.33 to −0.36) and −1.45 (CrI −1.91 to −0.98), respectively. This suggests no evidence of inconsistency but highlights that EMMELKAMP2006’s findings diverge from the rest of the evidence, as it suggests a negative effect on patient recovery, where all other estimates indicate positive effects. We would advise that these studies (including studies in the same evidence loops as EMMELKAMP2006) be double checked and a decision made as to their inclusion. If there are no data extraction errors or there is no reason identified to exclude them, then because there was no evidence of inconsistency in the global and local checks, it may be appropriate to proceed to step 3 of the model selection process. However, results should be interpreted with caution regarding the potential for these outliers to influence the conclusions of the analysis, particularly for loops of treatments including EMMELKAMP2006, and sensitivity analyses excluding these studies would be advised.

#### Step 3: Class effects assessment

In this step, we assess whether a class assumption is suitable for the data.

Table 4 shows that the exchangeable class model has an improved model fit compared with the no-class model selected at step 1, with a lower DIC (251.1 v. 257.0). This reduction in DIC by 5.9 points is due to the exchangeable class model giving a similar absolute fit to the no-class model (residual deviance 162.5 compared with 163.3) but reduced model complexity (effective number of parameters, $p_{D}$ = 94.9 in the no-class model compared with 87.8 in the exchangeable class model). Furthermore, the between-study standard deviation is similar in the exchangeable class and no-class models; we would have been concerned if this increased with the inclusion of class effects.

**Table 4:** Residual Deviance, $p_{D}$ , DIC, and Posterior Mean and 95% Credible Interval for τ for the No Class Effect and Exchangeable Class Models, Both with Random Effects

Model	Treatment Effect	Residual Deviance	$p_{D}$	DIC	τ
No class effect	Random	162.5	94.9	257.4	0.21 (0.15–0.27)
Exchangeable class	Random	163.3	87.8	251.1	0.20 (0.14–0.26)

DIC, deviance information criterion.

**Figure 7:**
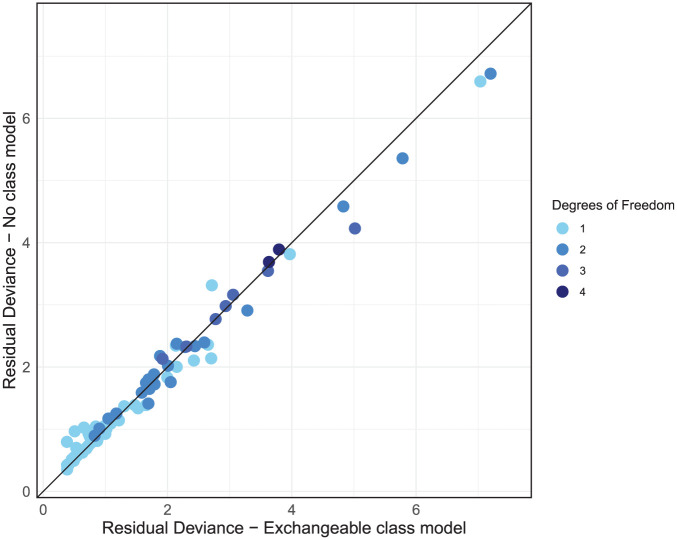
Residual deviance comparison between exchangeable class and no-class models, both with random effects.

Examining the residual deviance contributions from both models on a dev-dev plot (Figure 7), we see that the data points all lie on the line of equality, indicating no concerns with the fit of specific data points in the exchangeable class model. In summary, the exchangeable class model provides a comparable absolute fit to the no-class model and does not inflate the between-study heterogeneity while reducing complexity and DIC. These findings suggest that a class model is appropriate for analyzing the social anxiety data.

#### Step 4: Class model assessment

After we have determined that a class effects model is appropriate, it is time to finalize which combination of common or exchangeable class effects and fixed or random treatment effects provides the most suitable model fit while considering between-study heterogeneity and within-class standard deviation. As we are using a RE model, we now fit 2 other models to our data: exchangeable class FE and common class RE. This gives us a total of 3 models to compare. From Table 5 we can see that model fit is better with the common and exchangeable class models with RE (posterior mean residual deviance of 158.6 and 163.3, respectively) compared with the exchangeable class model with FEs (284.6). The between-study heterogeneity is higher for the common class model (0.25, 95% Crl: 0.20 to 0.31), compared with the exchangeable class model (0.20, 95% Crl: 0.14 to 0.26). This difference in between-study heterogeneity underscores the tradeoffs inherent in model selection: while the common class model offers a marginally better fit according to residual deviance, it does so at the expense of increased heterogeneity. When absolute fit is penalized for complexity, the common and exchangeable class models with RE give similar fit (DIC of 252.1 and 251.1, respectively) and are substantially preferable to the exchangeable class model with FE (318.7).

**Table 5:** Residual Deviance, $p_{D}$ , DIC, and τ Comparison between the Common Class Model with Random Effects against 2 Exchangeable Class Models, one with Random Effects and the Other with Fixed Effects

Model	Treatment Effects	Residual Deviance	$p_{D}$	DIC	τ
Common class	Random	158.6	93.5	252.1	0.25 (0.20–0.31)
Exchangeable class	Random	163.3	87.8	251.1	0.20 (0.14–0.26)
Exchangeable class	Fixed	284.6	34.1	318.7	—

DIC, deviance information criterion.

**Figure 8:**
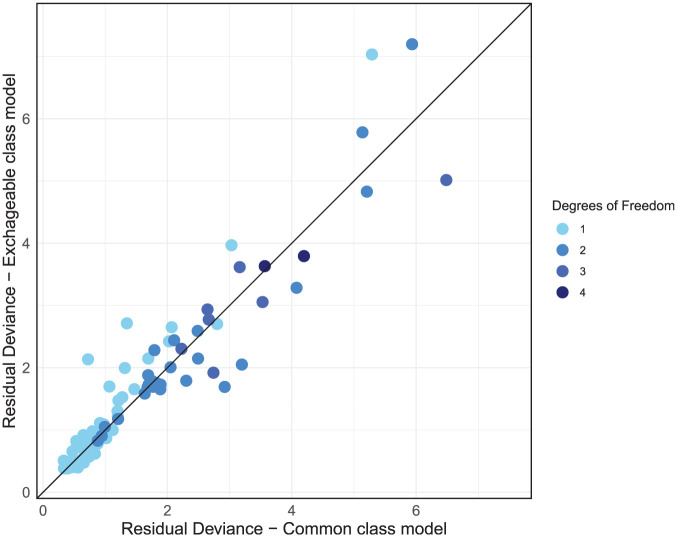
Residual deviance comparison between exchangeable class and common class models, both with random effects.

The dev-dev plot (Figure 8) comparing the exchangeable class and common class models with RE reveals a cluster of data points in the lower left corner of the plot that are fit well by both models with low residual deviances. As we move away from this cluster, points disperse further from the line of equality, albeit in a somewhat even distribution above and below it. This suggests that while some data points are better fit by the common class model, others are better fit by the exchangeable class model.

Given that the DIC scores are very similar between the RE common class and RE exchangeable class models, with the common class model giving slightly better absolute fit and the exchangeable class having less model complexity with lower heterogeneity, either model could be used here, and the final selection should largely be driven by clinical interpretability and the decision context. If decision makers are interested in the effects of individual treatments or are concerned with the variability of treatment effects within classes, then the exchangeable class model is more suitable. If decision makers wish to make recommendations for treatment classes and are not concerned with potential variability of treatment effects within classes, then the common class model may be preferred, as class-level effects and rankings are estimated with more precision, as shown in Figures 4 and 5.

**Figure 9:**
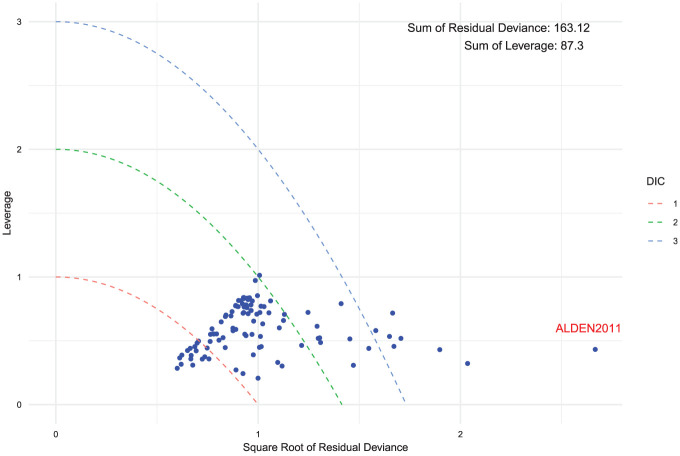
Leverage versus square root of the residual deviance for the exchangeable class random-effects model. Curves of $x^{2}+y=c$ where c=1,2,3 represent constant contributions to the deviance information criterion (DIC). The totals for residual deviance and leverage across all data points are indicated in the annotation.

After selecting our final preferred model, we performed a model fit check using a leverage plot. We adjusted the leverage and residual deviance by dividing them by their respective degrees of freedom. This ensures that studies with multiple arms do not exhibit disproportionately higher residual deviance or leverage compared with those with fewer arms. From Figure 9, all points are close to or below 1 on the leverage scale, indicating that no single study has an undue influence on the model fit. We also observe that there are a number of data points that exceed the c=3 threshold due to high residual deviance. Although our previous steps have already confirmed that data points with elevated residual deviance do not warrant exclusion, ALDEN2011 stands out. Since ALDEN2011 has undergone the standard checks in previous steps without raising concerns, there is no immediate indication to exclude it. That said, if any data points were found to have underlying issues, removing them would require returning to step 1 and refitting the model on the revised dataset. If no points are removed, this model remains our final choice, and we conclude the analysis here.

## Discussion

In this article, we have described a general framework for class effect NMA models, outlined the key assumptions required for their application, and introduced a model selection strategy designed to streamline the use of class effects in NMA. To our knowledge, this represents the first comprehensive guide on the use and selection of class effects models within NMA. We applied this model selection strategy to the social anxiety example, demonstrating the step-by-step approach to assess the range of potential assumptions and models for NMA when treatments can be grouped into classes.

Class effects models are widely used in the literature. However, many previous analyses^14,17,25^ that used class effects models did not assess the class assumption or evaluated only 1 type of class assumption. Although the literature has guidelines for testing consistency and heterogeneity,^1,21,26^ there has been little guidance on how to apply this with class effect models.

The only other class model selection strategy we know of proposes judging model fit by whether the posterior mean residual deviance ( $D_{\mathrm{res}}$ ) is “close” to the number of data points,^27^ but it does not define what “close” means. In our social anxiety example, this ambiguity made the rule difficult to apply consistently, and different reasonable interpretations of “close” could have led to different final models. Moreover, that approach does not set out a clear rationale for when and why a class model should be used, a choice that will influence the model selected. In contrast, our strategy provides explicit numerical guidance for model comparison and incorporates dedicated checks for heterogeneity (step 1) and inconsistency (step 2) before assessing class assumptions. By starting with the simplest no-class model and layering assumptions only when supported by evidence, our framework delivers a transparent, reproducible pathway for selecting class-effect models that is grounded in both statistical diagnostics and practical decision needs.

While our model selection strategy relies on statistical criteria such as DIC and residual deviance, these alone may not be sufficient to distinguish between competing models in smaller datasets, for example in health technology assessment, where information is often more limited. In such cases, clinical plausibility becomes critical. Where the data do not provide enough information to clearly favor a class effect model over a nonclass model, decisions on whether to pool should be guided by prior clinical knowledge about the likely similarity of treatment effects within a class. This reflects the reality that, in many applied settings, model choice is constrained by the quantity and quality of available evidence, and the best-fitting model in statistical terms may still be unsuitable if it conflicts with strong prior clinical beliefs or lacks adequate data support.

The importance of the plausibility of the class effect and the assumptions regarding it is highlighted by the fact that, even if 2 models have similar fit, the preferred model may not always be the simplest. For example, an exchangeable class effect FE model that has strong clinical rationale may still be preferred to an RE model that does not model class, because the exchangeable class model better partitions the different parts of the model that are likely to be contributing to the variance. This therefore may lead to more appropriate descriptions of uncertainty, even if the RE model is a simpler model.

Recent work by Nikolaidis et al.^28^ situates class effect models within a broader taxonomy of information-sharing approaches used in health technology assessment, distinguishing 4 “core” relationships: functional (lumping), exchangeability based, prior based, and multivariate information sharing. The hierarchical class models we present fall in the exchangeability-based category: treatment effects in the same class are assumed exchangeable draws from a class-level distribution, so information is shared through partial shrinkage. The amount of sharing is governed by the within-class heterogeneity ( $\sigma_{c}$ ). As $\sigma_{c}\to 0$ , the model behaves like full lumping (common class), whereas large $\sigma_{c}$ approaches a no-class (“splitting”) model. Posterior uncertainty in $\sigma_{c}$ therefore places the realized borrowing somewhere between these extremes, typically yielding less sharing than the prior mean would suggest.^28^ While such exchangeability models describe heterogeneity within classes, they do not explain it. If substantive knowledge suggests an ordered structure (eg, escalating doses or clearly graded potencies), a functional, dose-response, or other structured model may be more appropriate.

Class effects are often used not only to analyze performance differences between classes but also to address the challenge of small sample sizes and sparse networks where they may improve precision. When there are many treatments, class effects models can simplify the range of decision options, with the average effectiveness of a class of treatments summarized by the class mean and the potential variability of treatment effects summarized by the class standard deviation. However, when choosing between different class effect models, it is important to consider the needs of the decision makers. The choice between an exchangeable or common class model is dictated by the research objectives, the clinical plausibility of such assumptions, and the statistical similarity of treatment effects within a class. Exchangeable class models are beneficial where it is important to distinguish the effects of individual treatments, for example, when recommendations and rankings are required for individual treatments but a no-class model is either not possible or very imprecise. Common class models are suitable for situations in which treatments within the same class show considerable homogeneity in their effects and mechanisms of action or when the primary interest lies only in estimating the class mean, regardless of within-class variability. This approach is useful when recommendations are intended for treatment classes as a whole rather than individual treatments within a class. However, using an exchangeable class model to distinguish between classes has important implications as the extra hierarchical level introduces an additional source of heterogeneity. As a result, the predictive distributions for different classes can overlap, making it difficult for decision makers to identify a clear rank of classes. Overlapping predictions may simply reflect genuine uncertainty in the available evidence. In such cases, the results still provide valuable information by accurately representing the underlying uncertainty and may indicate that further evidence is needed before strong recommendations can be made. Careful consideration of model choice is therefore important, as selecting a more complex model than necessary could unnecessarily increase uncertainty, whereas an oversimplified model may fail to reflect real heterogeneity.

In the social anxiety example, we used an informative prior distribution for the class standard deviations following the original analysis.^17^ This resulted in similar class standard deviations across different classes, because the prior distribution was strongly informative for most of the classes. A potential alternative would have been to share class standard deviations across groups of similar classes in the network to help estimate the model without requiring informative priors. In our view, this approach would be preferred as long as the assumption of common class standard deviations is plausible across the chosen classes. A crucial step in this process would involve working closely with clinicians to get their expert views on the clinical similarities and differences between treatments and treatment classes. Clinicians can offer valuable information on whether the assumption of common class standard deviations is likely to hold based on therapeutic mechanisms, expected treatment effects, or other biological factors. Their input would guide the decision on sharing class standard deviations, ensuring that the statistical assumptions align with real-world clinical practice.

Mixed-class models (in which different class assumptions are used for different classes) can be incorporated into our model selection strategy, but there needs to be a clear justification for their use. Such models may be relevant if prior clinical knowledge suggests that specific treatment classes warrant distinct assumptions or if particular issues arise during model selection, although these cases are expected to be infrequent. For instance, in step 3, if the dev-dev plot indicates that most data points within a particular class are outliers below the line of equality, a mixed-class modeling approach might be justified. In this context, reassessment and potential “unclassing” of the outlier class could be considered, meaning the class effect assumption is removed and its treatments are modeled independently, provided there is sufficient supporting evidence and clinical rationale. Similarly, in step 4, if data points within a class consistently align with an alternative model that is not the preferred one, a mixed-class model approach may more accurately represent that class. Even in such cases, there needs to be sufficient improvement in model fit criteria to justify the added complexity of a mixed-class model. Overall, a unified class assumption simplifies model interpretation, reducing complexity and ensuring clarity in the conclusions.

Where results from class models are used to inform a health economic model, consideration needs to be given as to which model inputs (costs, adverse events, efficacy) are treatment specific and which are common for treatments in a class. For example, efficacy may come from a common class effect model, but costs and adverse events may differ between treatments. Thus, all individual treatments can be included in an economic model even when efficacy comes from the common class model. If an exchangeable class model is fitted, then the treatment-specific efficacy estimates can also be used. If decisions are to be made at the class level, then the estimate of efficacy for the class can be used from either the common class or exchangeable class model, but to estimate a typical class, cost assumptions about the likely market share of treatments within that class would be required to obtain an average class cost. Alternatively, scenarios could be run using the cheapest or most expensive treatment within the class. These complexities may have substantial effects on the resulting cost-effectiveness estimates informed by a class effect model. Nonetheless, health economic models typically require costing and evaluating the actual treatments rather than the average class effects, as decision makers need to account for the specific cost-effectiveness of individual interventions. Therefore, while common class models provide useful generalizations, they may not be suitable for health economic evaluations, in which the precision of costing individual treatments is critical.

The multinma package also implements multilevel network meta-regression (ML-NMR) to combine individual participant data and aggregate data, performing population adjustment to account for differences between study populations and obtain estimates in specific target populations. We did not make use of this functionality here as we did not have access to any individual level patient data (IPD) but the capability to incorporate class effects within ML-NMR models offers exciting opportunities for future research. Incorporating individual-level covariates within a class effects model may increase the feasibility of the class effects assumptions, while the inclusion of class effects in ML-NMR may help to increase precision. Furthermore, we plan to use both class effects and ML-NMR to address situations involving disconnected networks of evidence, thereby broadening the applicability of these approaches. Future research could also consider incorporating threshold analysis methods, as demonstrated by Phillippo et al.,^29^ and using established software implementations such as the CINeMA package (https://cinema.ispm.unibe.ch/) to assess the robustness of decisions in class-effect NMA.

In conclusion, our model selection strategy offers a practical tool for researchers and decision makers aiming to incorporate class effects into NMA. It provides comprehensive guidance on the range of assumptions made by such methods and outlines how these may be assessed, helping users choose the most suitable model based on expert knowledge, the decision context, and statistical considerations. Throughout, we have underscored the importance of comprehensive clinical understanding of the treatments included and the validity of their grouping into distinct classes. Furthermore, this work lays the foundation for exploring the use of class effects in disconnected networks, paving the way for future research in this area. By incorporating class effects into the multinma package, we have made this approach more accessible to users who want to perform NMA or ML-NMR with class effects.

## Supplemental Material

sj-docx-1-mdm-10.1177_0272989X251389887 – Supplemental material for Network Meta-Analysis with Class Effects: A Practical Guide and Model Selection Algorithm Supplemental material, sj-docx-1-mdm-10.1177_0272989X251389887 for Network Meta-Analysis with Class Effects: A Practical Guide and Model Selection Algorithm by Samuel J. Perren, Hugo Pedder, Nicky J. Welton and David M. Phillippo in Medical Decision Making
