# Supplementary material for: Network Meta-Analysis with Class Effects: A Practical Guide and Model Selection Algorithm
Source: Med Decis Making. 2025 Nov 8;46(3):275–95. doi: 10.1177/0272989X251389887 (PMC12976104; doi:10.1177/0272989X251389887)
Supplement: sj-docx-1-mdm-10.1177_0272989X251389887 – Supplemental material for Network Meta-Analysis with Class Effects: A Practical Guide and Model Selection Algorithm [file sj-docx-1-mdm-10.1177_0272989X251389887.docx]

### 8 Appendix

Table S1: Treatment and Class Relative Effects versus Waitlist in No class, Common Class, and Exchangeable Models with Random Effects. Details the number of trials and participants, and presents the standard mean difference (SMD) with 95% Credible intervals for each model

| Treatment | Trials | Participants | No class Model | Common Class | Exchangeable Class |
| --- | --- | --- | --- | --- | --- |
| **Controls** | | | | | |
| Waitlist | 28 | 802 | Reference | Reference | Reference |
| Placebo Pill | 42 | 3623 | -0.47 (-0.73 to -0.21) | -0.41 (-0.67 to -0.15) | -0.44 (-0.69 to -0.20) |
| Psychological Placebo | 6 | 145 | -0.68 (-0.97 to -0.38) | -0.57 (-0.90 to -0.27) | -0.65 (-0.94 to -0.37) |
| **Pharmacological interventions** | | | | | |
| Anticonvulsants | 5 | 242 | - | -0.70 (-1.07 to -0.32) | -0.78 (-1.32 to -0.23) |
| Gabapentin | 1 | 34 | -0.97 (-1.61 to -0.34) | - | -0.85 (-1.39 to -0.33) |
| Levetiracetam | 1 | 9 | -0.86 (-1.82 to 0.11) | - | -0.79 (-1.46 to -0.13) |
| Pregabalin | 3 | 199 | -0.70 (-1.09 to -0.31) | - | -0.69 (-1.04 to -0.34) |
| Benzodiazepines | 5 | 112 | - | -0.96 (-1.33 to -0.59) | -0.94 (-1.53 to -0.30) |
| Alprazolam | 1 | 12 | -0.76 (-1.43 to -0.12) | - | -0.84 (-1.38 to -0.30) |
| Clonazepam | 4 | 100 | -1.10 (-1.53 to -0.68) | - | -1.06 (-1.43 to -0.69) |
| Monoamine oxidase inhibitors | 11 | 615 | - | -0.94 (-1.22 to -0.66) | -0.99 (-1.56 to -0.42) |
| Moclobemide | 6 | 490 | -0.70 (-1.03 to -0.37) | - | -0.71 (-1.01 to -0.42) |
| Phenelzine | 5 | 125 | -1.35 (-1.67 to -1.04) | - | -1.28 (-1.58 to -0.96) |
| Noradrenergic and specific serotonergic antidepressants | 1 | 30 | - | -0.75 (-1.47 to -0.03) | -0.77 (-1.54 to 0.02) |
| Mirtazapine | 1 | 30 | -0.80 (-1.49 to -0.15) | - | -0.77 (-1.47 to -0.10) |
| SSRIs and SNRIs | 32 | 4043 | - | -0.88 (-1.15 to -0.61) | -0.89 (-1.19 to -0.59) |
| Citalopram | 2 | 18 | -0.70 (-1.30 to -0.10) | - | -0.84 (-1.22 to -0.44) |
| Escitalopram | 2 | 675 | -0.87 (-1.23 to -0.52) | - | -0.86 (-1.17 to -0.55) |
| Fluoxetine | 3 | 107 | -0.83 (-1.16 to -0.49) | - | -0.86 (-1.15 to -0.56) |
| Fluvoxamine | 5 | 500 | -0.94 (-1.27 to -0.59) | - | -0.90 (-1.22 to -0.61) |
| Paroxetine | 12 | 1449 | -1.01 (-1.30 to -0.71) | - | -0.96 (-1.23 to -0.69) |
| Sertraline | 3 | 535 | -0.91 (-1.26 to -0.57) | - | -0.89 (-1.20 to -0.58) |
| Venlafaxine | 5 | 759 | -0.95 (-1.26 to -0.62) | - | -0.91 (-1.20 to -0.62) |
| **Psychological and behavioural interventions** | | | | | |
| Exercise promotion (class) | 1 | 18 | - | -0.24 (-1.07 to 0.58) | -0.18 (-1.18 to 0.87) |
| Exercise promotion | 1 | 18 | -0.18 (-1.06 to 0.66) | - | -0.17 (-0.96 to 0.64) |
| Exposure and social skills | 10 | 227 | - | -0.77 (-1.05 to -0.50) | -0.83 (-1.43 to -0.23) |
| Exposure in vivo | 9 | 199 | -0.83 (-1.09 to -0.57) | - | -0.82 (-1.08 to -0.57) |
| Social skills training | 1 | 28 | -0.84 (-1.49 to -0.16) | - | -0.83 (-1.39 to -0.28) |
| Group CBT | 28 | 984 | - | -0.78 (-0.97 to -0.60) | -0.88 (-1.30 to -0.48) |
| Heimberg model | 11 | 338 | -0.81 (-1.05 to -0.57) | - | -0.78 (-1.01 to -0.56) |
| Other (no model specified) | 16 | 583 | -0.81 (-1.01 to -0.60) | - | -0.82 (-1.01 to -0.63) |
| Enhanced CBT | 1 | 63 | -1.17 (-1.65 to -0.66) | - | -1.05 (-1.47 to -0.64) |
| Individual CBT | 15 | 562 | - | -1.19 (-1.39 to -0.99) | -1.18 (-1.58 to -0.80) |
| Hope, Heimberg, and Turk model | 2 | 53 | -0.93 (-1.41 to -0.44) | - | -1.03 (-1.43 to -0.62) |
| Other (no model specified) | 6 | 163 | -1.14 (-1.50 to -0.80) | - | -1.18 (-1.49 to -0.89) |
| Clark and Wells cognitive therapy model | 3 | 97 | -1.62 (-1.97 to -1.29) | - | -1.53 (-1.84 to -1.22) |
| Clark and Wells cognitive therapy shortened sessions | 4 | 249 | -0.95 (-1.23 to -0.68) | - | -0.99 (-1.24 to -0.75) |
| Other psychological therapy | 7 | 182 | - | -0.40 (-0.75 to -0.04) | -0.34 (-0.81 to 0.16) |
| Interpersonal psychotherapy | 2 | 64 | -0.43 (-0.88 to 0.02) | - | -0.42 (-0.82 to -0.02) |
| Mindfulness training | 3 | 64 | -0.35 (-0.86 to 0.17) | - | -0.33 (-0.79 to 0.11) |
| Supportive therapy | 2 | 54 | -0.18 (-0.72 to 0.36) | - | -0.25 (-0.69 to 0.22) |
| Psychodynamic psychotherapy (class) | 3 | 185 | - | -0.67 (-1.03 to -0.29) | -0.62 (-1.32 to 0.10) |
| Psychodynamic psychotherapy | 3 | 185 | -0.60 (-0.92 to -0.26) | - | -0.63 (-0.94 to -0.30) |
| Self-help with support | 16 | 748 | - | -0.85 (-1.04 to -0.66) | -0.84 (-1.35 to -0.33) |
| Book with support | 3 | 52 | -0.84 (-1.22 to -0.45) | - | -0.83 (-1.16 to -0.50) |
| Internet with support | 13 | 696 | -0.85 (-1.02 to -0.67) | - | -0.85 (-1.02 to -0.68) |
| Self-help without support | 9 | 406 | - | -0.68 (-0.92 to -0.44) | -0.67 (-1.16 to -0.15) |
| Book without support | 4 | 136 | -0.75 (-1.03 to -0.47) | - | -0.73 (-0.98 to -0.49) |
| Internet without support | 5 | 270 | -0.59 (-0.89 to -0.29) | - | -0.60 (-0.90 to -0.32) |
| **Combined interventions** | | | | | |
| Combined | 5 | 156 | - | -1.21 (-1.54 to -0.90) | -1.28 (-1.73 to -0.82) |
| Group CBT and moclobemide | 1 | 22 | -1.14 (-1.77 to -0.53) | - | -1.21 (-1.69 to -0.69) |
| Group CBT and fluoxetine | 1 | 59 | -0.80 (-1.22 to -0.38) | - | -0.91 (-1.31 to -0.52) |
| Group CBT and phenelzine | 1 | 32 | -1.93 (-2.40 to -1.44) | - | -1.71 (-2.15 to -1.26) |
| Psychodynamic and clonazepam | 1 | 29 | -1.22 (-1.98 to -0.42) | - | -1.21 (-1.78 to -0.63) |
| Paroxetine and clonazepam | 1 | 14 | -1.44 (-2.27 to -0.62) | - | -1.32 (-1.92 to -0.72) |

Figure S1: Rank probability of treatments across NMA and Exchangeable class models employing random treatment effects

| Model | Treatment effect | Residual deviance | pD | DIC | tau |
| --- | --- | --- | --- | --- | --- |
| UME | Fixed | 285.2 | 60.3 | 345.5 | NA |
| UME | Random | 161.4 | 109.1 | 270.4 | 0.22 (0.16 - 0.29) |
| NMA | Fixed | 288.3 | 40.1 | 328.4 | NA |
| NMA | Random | 162.5 | 94.9 | 257.4 | 0.21 (0.15 - 0.27) |
| Common Class Effects | Fixed | 377.2 | 16.2 | 393.4 | NA |
| Common Class Effects | Random | 158.6 | 93.5 | 252.1 | 0.25 (0.20 - 0.31) |
| Exchangeable Class Effects | Fixed | 284.4 | 34.1 | 318.7 | NA |
| Exchangeable Class Effects | Random | 163.3 | 87.8 | 251.1 | 0.20 (0.14 - 0.26) |

Table S2: Comparison of UME, NMA, Common Class, and Exchangeable Class Models with Fixed and Random Effects. Displays Residual Deviance, pD, DIC, and $\tau$ for random effect models, facilitating model performance and consistency analysis

Figure S2: Comparison of Median Treatment Ranks with 95% Credible Intervals for Exchangeable Class and No Class Models Using Random Treatment Effects, Grouped by Treatment Class.

Figure S3: Comparison of Median Treatment Ranks with 95% Credible Intervals for Exchangeable Class and Common Class Models Using Random Treatment Effects.

| Exchangeable Class | Random | Fixed | n |
| --- | --- | --- | --- |
| Anticonvulsants | 0.30 (0.10 - 0.50) | 0.31 (0.12 - 0.50) | 3 |
| Benzodiazepines | 0.32 (0.14 - 0.51) | 0.32 (0.14 - 0.51) | 2 |
| CBT group | 0.30 (0.11 - 0.49) | 0.30 (0.12 - 0.50) | 3 |
| CBT individual | 0.33 (0.17 - 0.50) | 0.33 (0.18 - 0.51) | 4 |
| Combined | 0.35 (0.20 - 0.52) | 0.37 (0.23 - 0.53) | 5 |
| Exercise and SH no support | 0.31 (0.13 - 0.50) | 0.30 (0.10 - 0.52) | 3 |
| Exposure | 0.31 (0.10 - 0.51) | 0.31 (0.10 - 0.51) | 2 |
| MAOI | 0.35 (0.19 - 0.53) | 0.35 (0.20 - 0.53) | 2 |
| SSRIs and NSSA | 0.16 (0.02 - 0.35) | 0.12 (0.02 - 0.32) | 8 |
| Psychodynamic and Other psychological therapies | 0.30 (0.11 - 0.49) | 0.29 (0.10 - 0.49) | 4 |
| Self-help with support | 0.30 (0.08 - 0.51) | 0.30 (0.10 - 0.50) | 2 |

Table S3: Within Class Standard Deviation in Exchangeable Class Effect Models (random and fixed effects), segmented by class (n = treatments per class))

| Code | Treatment | Class |
| --- | --- | --- |
| 1 | Waitlist | Waitlist |
| 2 | Pill placebo | Pill placebo |
| 3 | Psychological placebo | Psychological placebo |
| 4 | Exercise promotion | Exercise promotion |
| 5 | Self help book no support | Self help no support |
| 6 | Self help internet no support |  |
| 7 | Self help book with support | Self help with support |
| 8 | Self help internet with support |  |
| 9 | Pregabalin | Anticonvulsants |
| 10 | Levetiracetam |  |
| 11 | Gabapentin |  |
| 12 | Mirtazapine | NSSA |
| 13 | Sertraline | SSRI/SNRI |
| 14 | Citalopram |  |
| 15 | Escitalopram |  |
| 16 | Fluoxetine |  |
| 17 | Fluvoxamine |  |
| 18 | Paroxetine |  |
| 19 | Venlafaxine |  |
| 20 | Alprazolam | Benzodiazepines |
| 21 | Clonazapam |  |
| 22 | Moclobemide | MAOI |
| 23 | Phenelzine |  |
| 24 | Exposure in vivo | Exposure |
| 25 | Social skills training |  |
| 26 | Supportive therapy | Other psychological therapies |
| 27 | Mindfulness |  |
| 28 | Interpersonal psychotherapy |  |
| 29 | Psychodynamic psychotherapy | Psychodynamic psychotherapy |
| 30 | CBT group | CBT group |
| 31 | CBT group Heimberg |  |
| 32 | CBT group Enhanced |  |
| 33 | Cognitive therapy shortened sessions | CBT individual |
| 34 | CBT individual Heimberg |  |
| 35 | CBT individual |  |
| 36 | Cognitive therapy |  |
| 37 | CBT group + Fluoxetine | Combined |
| 38 | Psychodynamic + Clonazepam |  |
| 39 | Paroxetine + Clonazapam |  |
| 40 | CBT group + Moclobemide |  |
| 41 | CBT group + Phenelzine |  |

Table S4: Treatment codes with their respective treatment names and what class of treatment they belong to
